# A disulfide bond A-like oxidoreductase is a strong candidate gene for self-incompatibility in apricot (*Prunus armeniaca*) pollen

**DOI:** 10.1093/jxb/erx336

**Published:** 2017-09-25

**Authors:** Juan Vicente Muñoz-Sanz, Elena Zuriaga, María L Badenes, Carlos Romero

**Affiliations:** 1 Fruit Tree Breeding Department. Instituto Valenciano de Investigaciones Agrarias (IVIA). CV-315, Km. 10, Moncada (Valencia), Spain; 2 Instituto de Biología Molecular y Celular de Plantas (IBMCP), Universidad Politécnica de Valencia-Consejo Superior de Investigaciones Científicas. C/Ingeniero Fausto Elio s/n, Valencia, Spain

**Keywords:** DsbA oxidoreductase, gametophytic self-incompatibility, *M*-locus, modifier, pollen-part mutation, *Prunus*

## Abstract

*S*-RNase based gametophytic self-incompatibility (SI) is a widespread prezygotic reproductive barrier in flowering plants. In the Solanaceae, Plantaginaceae and Rosaceae gametophytic SI is controlled by the pistil-specific S-RNases and the pollen *S*-locus F-box proteins but non-*S*-specific factors, namely modifiers, are also required. In apricot, *Prunus armeniaca* (Rosaceae), we previously mapped two pollen-part mutations that confer self-compatibility in cultivars Canino and Katy at the distal end of chromosome 3 (*M*-locus) unlinked to the *S*-locus. Here, we used high-resolution mapping to identify the *M*-locus with an ~134 kb segment containing *ParM-1*–*16* genes. Gene expression analysis identified four genes preferentially expressed in anthers as modifier gene candidates, *ParM-6*, *-7*, *-9* and *-14*. Variant calling of WGS Illumina data from Canino, Katy, and 10 self-incompatible cultivars detected a 358 bp miniature inverted-repeat transposable element (MITE) insertion in *ParM-7* shared only by self-compatible apricots, supporting *ParM-7* as strong candidate gene required for SI. *ParM-7* encodes a disulfide bond A-like oxidoreductase protein, which we named ParMDO. The MITE insertion truncates the *ParMDO* ORF and produces a loss of SI function, suggesting that pollen rejection in *Prunus* is dependent on redox regulation. Based on phylogentic analyses we also suggest that *ParMDO* may have originated from a tandem duplication followed by subfunctionalization and pollen-specific expression.

## Teaser

A disulfide bond A-like oxidoreductase encoded by a pollen modifier candidate gene is required for gametophytic self-incompatibility in apricot (*Prunus armeniaca*) as supported by loss-of-function mutations.

## Introduction

*S*-RNase-based self-incompatibility (SI) is found in diverse plant families, including Rosaceae, Solanaceae and Plantaginaceae (Igic and Kohn, 2001). The multiallelic *S*-locus encodes at least two genes functioning as female and male *S*-specificity determinants (de Nettancourt, 2001). Pollen is rejected when its *S*-haplotype is the same as either of the two *S*-haplotypes present in the style. In *Prunus* (Rosaceae) the *S-RNase* gene is the stylar specificity factor (McClure *et al.*, 1989) and an F-box protein gene, *S*-locus F-box (*SFB*), encodes the pollen determinant (Lai *et al.*, 2002; Ushijima *et al.*, 2003).

The stylar S-RNase proteins are thought to specifically recognize and reject self-pollen by virtue of their cytotoxic activity, while nonself pollen is not inhibited (Huang *et al.*, 1994). Although it is unclear how self *S*-RNases and F-box proteins interact, in Solanaceae, the F-box proteins likely form a conventional Skp1-Cul1-F-box-protein (SCF) E3 ubiquitin ligase complex that recognizes nonself S-RNases, promoting ubiquitination and subsequent degradation by the 26S proteasome (Qiao *et al.*, 2004; Hua *et al.*, 2006). The collaborative nonself-recognition model for SI in Solanaceae is a refinement of this degradation model that postulates that multiple *S*-locus F-box (SLF) proteins work together to recognize nonself *S*-RNases (Kubo *et al.*, 2010). However, these models do not account for uptake of S-RNase into the lumen of the endomembrane system or the requirement for modifier genes (Goldraij *et al.*, 2006). The basics of the *S*-RNase-based SI system are preserved in Solanaceae, Plantaginaceae, and *Pyreae* (Rosaceae), but *Prunus* (Rosaceae) exhibits distinct genetic and molecular features. Remarkably, loss-of-function pollen-part mutations (PPMs) that truncate *Prunus S*-locus F-box (*SFB*) genes cause self-compatibility (SC), which is opposite to the expectation from the collaborative nonself recognition model (Tao and Iezzoni, 2010). Matsumoto and Tao (2016) recently proposed a different model in *Prunus* where self-SFB protects self-*S*-RNases from a ‘general inhibitor’, proposed to be the *S*-locus linked SLF-like2 factor, which detoxifies all self/nonself-*S*-RNases in pollen tubes.

*S*-locus unlinked genes providing a function other than *S*-specificity, namely modifier genes, are also required for SI (McClure *et al.*, 2000). Thus, identifying and characterizing SI modifier genes is also especially important for understanding *Prunus* SI. Particularly in pollen, modifiers were firstly identified in *Petunia* and *Nicotiana* (Solanaceae) including the components of the SCF E3 ubiquitin ligase complex PhSSK1 (Zhao *et al.*, 2010), Rbx1, and Cullin (Li *et al.*, 2014). Molecular level studies also have identified putative pollen modifiers in some rosaceous species. In *Malus* (Rosaceae), an ATP binding cassette subfamily F (ABCF) transporter interacts with *S*-RNases and has been proposed to facilitate transport into pollen tubes (Meng *et al.*, 2014) and *Skp1* and *Cullin1* orthologs have also been found in *Prunus* (Matsumoto *et al.*, 2012). However, no functional studies have tested whether the corresponding genes are required for SI *in vivo* because fruit trees are not well suited for reverse genetics.

Forward genetic studies in sweet cherry (*Prunus avium* L.) and apricot (*Prunus armeniaca* L.) have identified non-*S*-locus PPMs conferring SC and therefore are predicted to affect SI modifier genes (Wünsch and Hormaza, 2004; Vilanova *et al.*, 2006). PPMs in apricot have been genetically characterized in detail in cultivars Canino and Katy. Genetic analyses show that Canino (*S*_2_*S*_C_*Mm*) carries two independent mutations conferring SC: an *SFB* gene insertion that disrupts the open reading frame (*S*_C_-haplotype) and an unlinked pollen modifier gene mutation (*m*-allele) (Vilanova *et al.*, 2006). Katy has a unique PPM in a modifier gene named *m*’ (*S*_1_*S*_2_*M*’*m*’) (Zuriaga *et al.*, 2013). Subsequent studies showed that Canino *m* and Katy *m’* map to overlapping regions at the distal end of chromosome 3, the *M*-locus (Zuriaga *et al.*, 2012; Zuriaga *et al.*, 2013), and are associated with the same haplotype (Muñoz-Sanz *et al.*, 2017). Thus, we hypothesized that Canino and Katy shared the same founder mutation, hereafter referred to as *m*.

Loss-of-function PPM *m* causes SC, so identifying the function of the corresponding mutated gene can provide mechanistic insights into *Prunus* SI and contribute to the knowledge of SI evolution in Rosaceae. In order to achieve this goal we developed two complementary strategies based on next generation sequencing (NGS) data: high-resolution mapping and a candidate gene approach.

## Materials and methods

### Plant material

Genomes and transcriptomes of the self-compatible apricot cultivars Canino (*S*_2_*S*_C_*Mm*) and Katy (*S*_1_*S*_2_*Mm*) and the self-incompatible Goldrich cultivar (*S*_1_*S*_2_*M*_1_*M*_2_) were analyzed by NGS. To fine-map the *M*-locus we used an outcross-type Goldrich×Canino (G×C) population consisting of 323 individuals, a Katy F_2_ (K×K) population consisting of 94 individuals, and 12 F_3_ populations derived from K×K-F_2_ individuals, ranging from *n*=2 to *n*=77 and totaling 344 seeds (Zuriaga *et al.*, 2012; Zuriaga *et al.*, 2013). Parents, recombinants, self-incompatible (SEO, Orange red, Harcot, Stella, Velázquez and Moniquí), and self-compatible apricots (Portici and Corbató) were surveyed for the presence of the *ParMDO* insertion. Canino×Canino (C×C)-F_2_ individuals CC-67 (*S*_2_*S*_2_*mm*) and CC-77 (*S*_C_*S*_C_*MM*) were used as controls. Most of these trees are maintained at the collection of the Instituto Valenciano de Investigaciones Agrarias (IVIA) in Valencia, Spain.

### Nucleic acid extraction and genotyping

Plant DNA isolation was performed according to Doyle and Doyle (1987). BAC DNA isolation was conducted by Macrogen Inc. Total RNA was extracted from leaves, petals, styles, ovaries, anthers, and pollen of balloon stage flowers using the RNeasy Plant Mini Kit (Qiagen, Hilden, Germany).

Simple sequence repeats (SSRs) were identified by RepeatMasker (Smit AFA, Hubley R & Green P. *RepeatMasker Open-4.0*. 2013–2015, http://www.repeatmasker.org). PCR amplifications were performed with a GeneAmp PCR System 9700 thermal cycler (Perkin-Elmer, Freemont, CA, USA) in a final volume of 20 µl, containing 75 mM Tris-HCl at pH 8.8, 20 mM (NH_4_)2SO_4_, 1.5 mM MgCl_2_, 0.1 mM of each dNTP, 20 ng of genomic DNA and 1 U of DreamTaq polymerase (Thermo Scientific, Waltham, MA), according to the procedure of Schuelke (2000) and using the following cycling conditions: 94°C for 2 min, then 35 cycles of 94°C for 45 s, 50–60°C for 1 min, and 72°C for 1 min and 15 s, finishing with 60°C for 30 min. Allele lengths were determined using an ABI Prism 3130 Genetic Analyzer with the aid of GeneMapper software, version 4.0 (Applied Biosystems, Foster City, CA, USA).

Single nucleotide polymorphisms (SNPs) and small insertions or deletions were called by CLC Genomics Workbench 8.0.1 (CLCbio, Qiagen) using the ‘basic variant detection’ tool and the alignments of cleaned Illumina reads of Canino, Katy, and Goldrich against the a*M*-supercontig (see below). PCR assays for SNP genotyping were performed using the same PCR cocktail described above for SSRs and the following cycling conditions: an initial denaturing step of 95°C for 2 min; 35 cycles of 95°C for 30 s, 52°C for 30 s and 72°C for 1 min; and a final extension of 72°C for 10 min. PCR products of four independent replicates were pooled, purified with a DNA Clean&Concentrator-5 Kit (Zymo Research, Irvine, CA) and sequenced by Sanger sequencing by Sistemas Genomicos S.L. (Valencia, Spain). All primers were designed with Primer3 v.0.4.0 (Untergasser *et al.*, 2012) (see Supplementary Table S2 at JXB online).

### Next generation sequencing

Apricot BAC clones (Vilanova *et al.*, 2003) were pyrosequenced by Macrogen Inc. (Seoul, South Korea) using 454 GS-FLX Titanium NGS (Roche). Whole genome sequencing (WGS) of Canino and Katy was conducted by Macrogen Inc. using Illumina HiSeq2000 paired-end (PE) reads and raw data were deposited in GenBank BioProject PRJNA360683. Goldrich and SEO WGS Illumina PE data were generated at genomic facilities at DHMRI (David H. Murdock Research Institution, Kannapolis, NC, USA; http://www.dhmri.org) and kindly provided by C. Dardick, T. Zhebentyayeva and A. Abbott. RNA sequencing (RNA-Seq) was performed using Illumina PE by the UCLA Neurosciences Genomic Core (University of California, USA). Two biological replicates were sampled from leaves and styles and three from anthers of Goldrich, Canino, and Katy (except for Katy styles; not available). Two technical replicates were performed per biological replicate (Supplementary Table S1). WGS reads of cultivars Orange red, Stella, Lambertin, Veecot, Harcot, Perfection, Moniquí, and Velázquez (Mariette *et al.*, 2016) were downloaded from the NCBI Sequence Read Archive (SRA) repository (see Supplementary Protocol S1 for details on NGS data pre-processing).

### Apricot *M*-locus (a*M*) supercontig *de novo* assembly

Cleaned 454 BAC sequences (Supplementary Table S1) were *de novo* assembled using CLC Workbench and the peach v1.0 and v2.0 (http://www.rosaceae.org) (Verde *et al.*, 2013) and *P. mume* (BioProject PRJNA171605) (Zhang *et al.*, 2012) genomes as references. BAC contigs were joined using *GAP4* (*Staden package*; staden.sourceforge.net) (Bonfield *et al.*, 1995) and synteny criteria (for more details see Supplementary Protocol S2). The final a*M*-supercontig sequence is deposited under GenBank accession number KY499716.

### Gene annotation and differential expression analysis

Goldrich, Canino, and Katy RNA-Seq data from leaf, style, and anther tissues were aligned to a modified peach genome created by replacing the peach *M*-locus region with the a*M*-supercontig sequence, between positions 18 380 006 and 18 815 966 at scaffold_3, using the ‘transcript discovery plug-in 2.0’ of CLC Genomics Workbench 8.0.1. Gene annotations were carried out with the CLC ‘transcript discovery’ tool and manually curated. Differential expression analysis was performed by aligning RNA-seq reads to the modified peach genome using Bowtie2 v2.2.4 (bowtie-bio.sourceforge.net/bowtie2) (Langmead *et al.*, 2009) through the Trinity software (Haas *et al.*, 2013). Transcript quantification was performed with RSEM (Li and Dewey, 2011) and the edgeR package (Robinson *et al.*, 2010) was used to call differentially expressed genes. A false discovery rate (FDR)≤0.05 was used to determine the threshold of the *P*-value in multiple tests (additional details are available in Supplementary Protocol S3). A heat map was generated using a custom R script.

RT-PCR analysis was performed using total RNA extracted from leaves, petals, ovaries, styles, and pollen from balloon stage flowers of Goldrich. Total cDNAs were synthesized using the PrimeScript RT reagent kit (Takara Bio, Otsu, Japan) with Oligo-d(T) primer. PCRs were performed using gene-specific primer sets (Supplementary Table S4) in a final volume of 20 μL containing 1×DreamTaq buffer, 0.2 mM of each dNTP, 250 μM of each primer, 1 U of DreamTaq DNA polymerase (Thermo Fisher), and 2 μL of cDNA template diluted 1:20 from the 10 μL synthesized using 500 ng of total RNA. Cycling conditions were as follows: an initial denaturing of 95°C for 2 min; 35 cycles of 95°C for 30 s, 55°C for 30 s and 72°C for 1 min; and a final extension of 72°C for 10 min (UNO96, VWR, Radnor, PA, USA). PCR products were electrophoresed in 0.8% (w/v) agarose gels stained with RedSafe nucleic acid staining solution (iNtRON Biotechnology, Korea) and visualized under UV light. Molecular sizes of amplified fragments were estimated using GeneRuler 100 bp Plus DNA ladder (Thermo Fisher).

### Variant calling and filtering

Illumina WGS cleaned reads from 12 apricot cultivars (Supplementary Table S1) were aligned to the modified peach genome using Bowtie2 v2.2.4 (Langmead *et al.*, 2009). Reads mapping to the ~134 kb annotated region, between positions 142 155 and 276 184 in the a*M*-supercontig, were realigned to call variants by CLC Genomics Workbench 6.0.1. Single nucleotide variants (SNVs) were called by the ‘quality-based variant detection’ tool and structural variants (SVs) by the ‘structural variation detection (beta) plug-in 3.0’ tool using default settings. Variants were sequentially filtered applying homemade Python scripts. Selected variants must be (i) heterozygous in Canino and Katy; (ii) absent in the 10 self-incompatible cultivars analyzed; and (iii) shared by Canino and Katy (Supplementary Table S5).

### *ParMDO* gene amplification and sequencing

*ParMDO m*- and *M*-alleles were PCR-amplified using CC-67, K06-17 (*mm*), CC-77 (*MM*) gDNAs, and CC-77 pollen cDNA as templates. Overlapping fragments comprising *ParMDO* were PCR-amplified with specific primer pairs (Supplementary Table S4) under the same conditions reported for RT-PCR (see ‘Gene annotation and differential expression analysis’ subsection in Materials and methods) and PCR products were purified (see the ‘Nucleic acids extraction and genotyping’ subsection in Materials & Methods). Fragments were sequenced by Sanger at the IBMCP Bioinformatics Service (http://www.ibmcp.upv.es), assembled with the *Staden package*, and deposited under GenBank accession numbers KY429940 and KY429941. The gene name was assigned according to the nomenclature detailed in the Gene Naming Guideline established by the Rosaceae Gene Name Standarization Subcommitee of RosEXEC/RosIGI (http://www.rosaceae.org). *ParMDO* insertion was PCR-genotyped using specific primers (Supplementary Table S4) and the same PCR conditions used for *ParMDO* amplification. PCR products were electrophoresed in 1% agarose gel.

### Ortholog analyses

ParMDO was BLASTed (Altschul *et al.*, 1990) against the NCBI nr protein database (Supplementary Table S4) and selected hits reciprocally BLASTed against the NCBI *Prunus* (taxid: 3754) protein database (Supplementary Table S6). Annotated genomes and predicted protein collections of *P. persica*, *M. domestica* (http://www.rosaceae.org), *S. lycopersicum* (http://solgenomics.net), and *A. thaliana* (htpp://www.arabidopsis.org) were used to identify *M*-locus syntenic blocks by BLASTP Reciprocal Best Hit (RBH) analysis of 62 predicted proteins encoded by genes located at the *Prunus persica* scaffold_3 (*M*-locus) (Supplementary Table S8). RBH analysis was carried out through custom-made Python scripts. Positive RBH anchors supporting syntenic blocks were visualized by Circos software (Krzywinski *et al.*, 2009). Phylogenetic tree-based analysis was conducted by MEGA6 software (Tamura *et al.*, 2013). Amino acid sequences (Supplementary Tables S6 and S7 and Supplementary Fig. S3) were aligned by ClustalW (Thompson *et al.*, 1994). Poorly aligned positions and divergent regions of the alignment were eliminated using Gblocks v.0.91b (Castresana, 2000). The best-fitting evolutionary model (LG+G), according to the Akaike information criterion (AIC), was implemented in the Maximum Likelihood (ML) phylogenetic analysis (Felsenstein, 1981) using 1000 bootstrap replications.

## Results

### The apricot *M*-locus

**Fig. 1.**
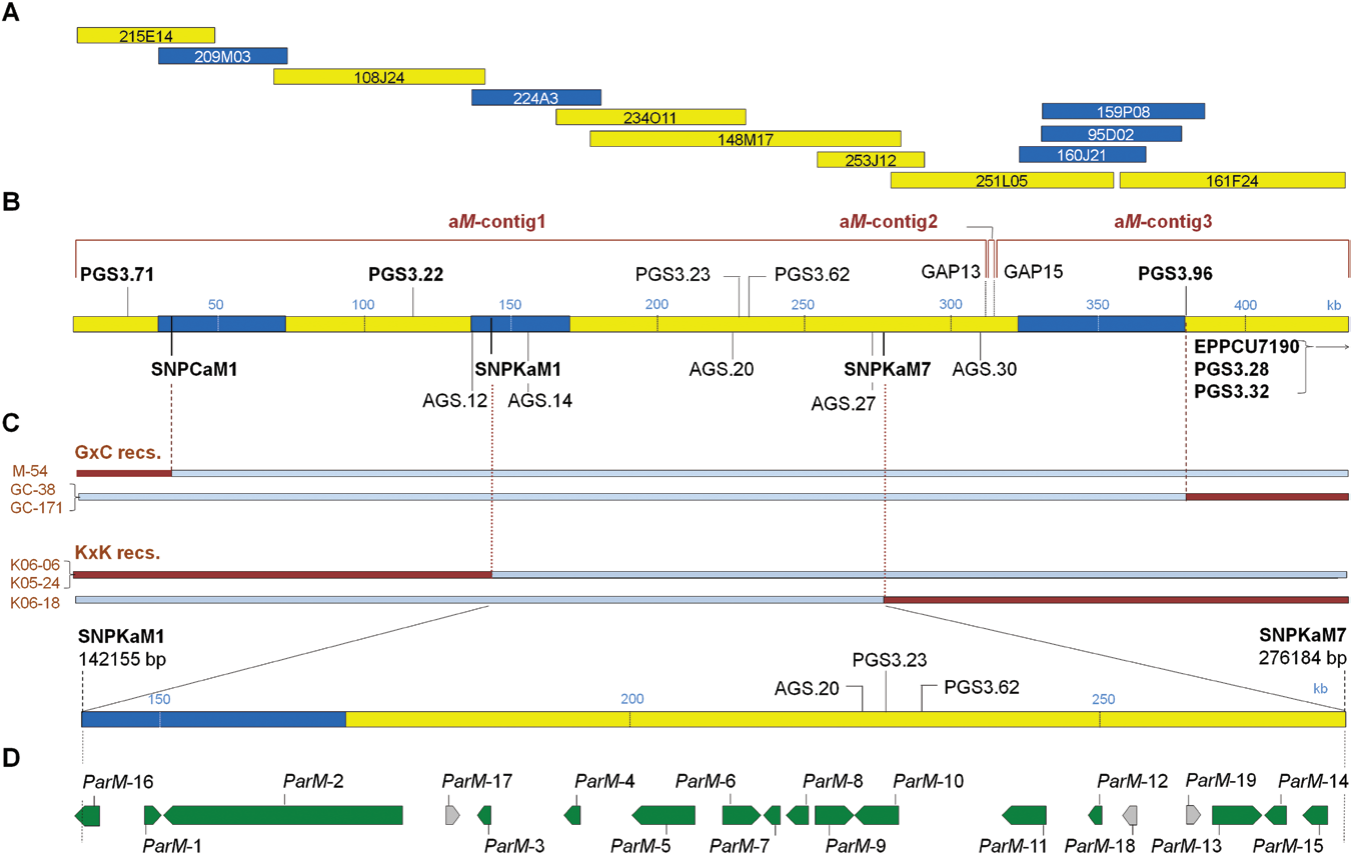
Genetic and physical maps of the apricot *M*-locus. (A) BAC clones from the self-incompatible cultivar Goldrich were used for *de novo M*-locus reference sequence assembly. BAC clones corresponding to the *M*_1_- and *M*_2_-haplotypes have a blue and yellow background, respectively. (B) Final a*M*-supercontig comprising major contigs a*M*-contig-1, -2, and -3 and the unresolved GAPs -13 and -15. Positions of peach (PGS3) and apricot (AGS) SSRs and SNPs are shown. (C) Genetic maps of the Canino and Katy *M*-loci were refined using new recombinants (left) and markers (*dashed* and *dotted lines*) delimiting a physical region of ~134 kb. (D) The ~134 kb region with the annotated apricot genes; green arrows, *ParM-1*–*16*. *ParMDO* corresponds to *ParM-7*. Grey arrows, *ParM-17* to *-19*. ORFs identified in *P. persica* and *P. mume* but not supported by *P. armenica* expression data.

PPM *m* conferring SC in Canino and Katy was previously mapped to overlapping intervals of 1.8 and 9.4 cM on chromosome 3 (*M*-locus), respectively (Zuriaga *et al.*, 2012; Zuriaga *et al.*, 2013). BAC clones from the self-incompatible apricot cultivar Goldrich covering the *M*-locus were anchored to the genetic maps and syntenic regions were identified in the peach (*Prunus persica* (L.) Batsch) genome (Zuriaga *et al.*, 2012). To facilitate identification of the pollen modifier mutation in the *M*-locus, we created an apricot reference sequence by pyrosequencing 12 BAC clones (Fig. 1; Supplementary Table S1). Most Goldrich BAC contigs anchored to the distal end of *P. persica* scaffold-3 (peach v1.0; http://www.rosaceae.org) (Verde *et al.*, 2013) between 18 380 and 18 816 kb. In total, 30 BAC contigs were located in the *M*-locus region. Five were assigned to the designated *M*_1_-haplotype and 25 to the *M*_2_-haplotype by SSR-genotyping (Fig. 1). BAC contigs were joined but two gaps, -13 and -15, could not be closed resulting in a physical map consisting of three contigs, namely 311 575 bp a*M*-contig1,3193 bp a*M*-contig2, and 120 995 bp a*M*-contig3, which were joined by Ns to form the 435 961 bp apricot *M*-locus supercontig, a*M*-supercontig (Fig. 1).

Genetic mapping studies defined the *M*-locus in Canino by the SSR flanking markers PGS3.71 and PGS3.96 (Zuriaga *et al.*, 2012) and these markers place the physical *M*-locus in a 361 960 bp interval within the a*M*-supercontig. Mapping in Katy located the *M*’-locus between PGS3.22 and EPPCU7190 markers, >1Mb according to the peach genome (Zuriaga *et al.*, 2013). The overlap of these two physical intervals places the *M*-locus in a 264 940 bp segment between PGS3.22 and PGS3.96 that contains *m* co-segregating SSR markers PGS3.62 and PGS3.23 (Fig. 1).

We generated new SSR and SNP markers and additional recombinants to further refine the genetic map. Forty SSRs were identified from the a*M*-supercontig (Supplementary Table S2) and thousands of variants were detected by mapping Goldrich, Canino, and Katy genomic Illumina reads to the a*M*-supercontig sequence, from which validated SNPs were selected (Supplementary Table S2). Only one SSR marker co-segregating with *m* (AGS.20) could be mapped in Canino. However, recombinant M-54 from the outcross population G×C revealed a breakpoint, corresponding to the SNPCaM1 marker, 15 kb downstream of PGS3.71. In Katy, five new SSRs were mapped using the F_2_ population K×K, identifying four recombinants between markers AGS.12 and AGS.30: K05-24, K06-06, K06-18, and K06-37. Additionally, 10 SNPs, including three between AGS.12 and AGS.14 and four between AGS.27 and AGS.30, were tested and recombination breakpoints were observed for SNPKaM1 and SNPKaM7, refining the *M*-locus to a physical interval of 134 030 bp. This overlapping region still contained markers tightly linked with *m* in mapping populations derived from Canino and Katy, namely PGS3.62, PGS3.23, and AGS.20 (Fig. 1, Supplementary Table S2, and Supplementary Fig. S1).

### *M*-locus genes preferentially expressed in pollen

RNA-Seq data from leaves, styles, and anthers of apricot cultivars Goldrich, Canino, and Katy were used for gene annotation and expression analyses (Supplementary Table S1). We created a modified peach reference genome by replacing the peach *M*-locus region with the a*M*-supercontig sequence and aligned trimmed reads from each cultivar. Gene annotation for the ~134 kb region was manually curated using the annotation from peach v1.0 and v2.0 (http://www.rosaceae.org) and *P. mume* (NCBI BioProject PRJNA171605) (Zhang *et al.*, 2012). A total of 15 full-length genes were identified and named *ParM-1*–*15* from the *Prunus armeniaca M*-locus (Fig. 1). Based on the position of the SNP KaM1 flanking marker another gene, *ParM-16*, was only partially included in the ~134 kb region. Three additional genes, *ParM-17*–*19* (grey arrows in Fig. 1), were consistently predicted in *P. persica* and *P. mume*, but were not supported by our apricot expression data (Supplementary Table S3). Apricot annotated genes showed high homology with the corresponding genes in the syntenic regions of *P. persica* and *P. mume*, greater than 98% identity in most cases (Supplementary Table S3).

**Fig. 2.**
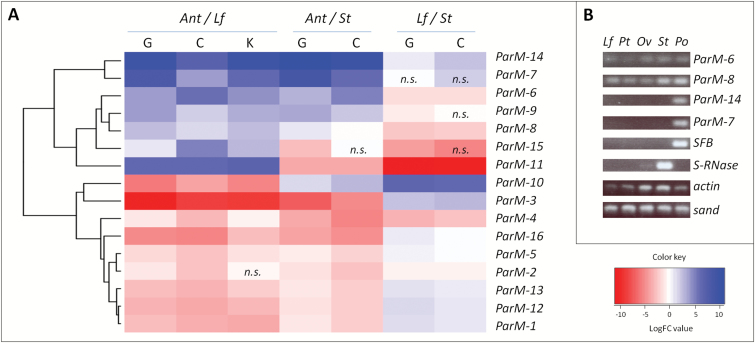
Gene expression analysis. (A) Heat map illustrating RNA-Seq differential expression data. Pairwise comparisons are shown for each apricot cultivar (columns). Blue, positive log fold-change (log FC) indicates higher expression in the first tissue compared with the second; red, negative log FC. Cultivars and RNA samples are as follows: G, Goldrich; C, Canino; K, Katy; Ant, Anther; St, Style; Lf, Leaf. Non-significant differences with *P*-values>0.001 are indicated (*n.s.*). (B) RT-PCR analysis comparing expression in different tissues from CC-77 (*MM*): *Lf*, leaf; *Pt*, petal; *Ov*, ovary; *St*, style; *Po*, pollen. Housekeeping genes *actin* and *sand-like* as well as style- and pollen-specific genes, *S-RNase* and *SFB*, respectively, were used as controls.

Fig. 2 shows that several *M*-locus genes are preferentially expressed in anthers. RNA-Seq data from leaves, styles, and anthers was used to interrogate *ParM-1*–*16* for differential expression. *ParM-6*, *-7*, *-8*, *-9*, *-11*, *-14*, and *-15* showed preferential expression in anthers compared with leaves in Canino, Goldrich, and Katy. Style data was not available for Katy, but four of these seven genes, *ParM-6*, *-7*, *-9* and *-14*, also showed preferential expression in anthers compared with styles in Canino and Goldrich (Fig. 2A). Particularly, *ParM-7* and *-14* showed strong expression in anthers, that is with a logFC~>5 across all samples. RT-PCR analyses confirmed that *ParM-7* and *ParM-14* are preferentially expressed in pollen displaying a pattern similar to the pollen-specific *SFB*. RT-PCR results further confirmed expression of *ParM-6* and *ParM-8* in pollen, but expression was also detected in leaf, petals, ovary, and style (Fig. 2B; Supplementary Fig. S2 and Supplementary Table S4).

### A 358 bp insertion in *ParM-7* explains loss of SI function in self-compatible apricot pollen

**Fig. 3.**
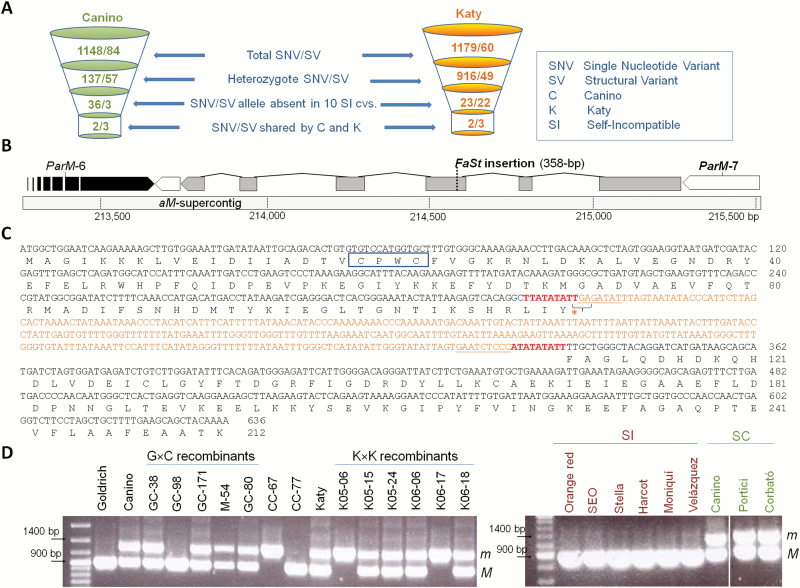
Identification of the variant causing SC in Canino and Katy pollen. (A) Variant filtering. SNVs and SVs within the a*M*-supercontig were called for Canino, Katy, and 10 self-incompatible cultivars (see Supplementary Table 1) and filtered as shown. (B) The insertional variant in *ParM-7*. *ParM-7* is transcribed from right to left. Grey, coding exons; white, UTR regions; black, *ParM-6* shown for reference; dashed line, 358 bp *FaSt* MITE insertion site. (C) *ParM-7* coding sequence and predicted amino acid sequence for *m*- and *M*-alleles. *FaSt* MITE insertion (orange) leads to a premature stop codon (asterisk) in the *m*-allele. *FaSt* MITE target site duplications and 5′ and 3′terminal inverted repeats are shown in red and underlined, respectively. The dicysteine redox motif CPWC conserved in DsbA-like proteins is boxed. (D) PCR-genotyping of *ParM-7* in mapping recombinants and self-compatible and self-incompatible apricot cultivars. Recombinants from G×C and K×K populations and homozygous controls CC-67 (*mm*) and CC-77 (*MM*) are included.

The ~134 kb *M*-locus region was interrogated for SNVs and SVs associated with *m* in Canino and Katy. Variant calling using Illumina reads from 12 apricots, including 10 self-incompatible cultivars (Supplementary Table S1), identified a total of 2831 variants; 1232 and 1239 in Canino and Katy, respectively (Fig. 3A and Supplementary Table S5). The genetics of SC in Canino and Katy imposed three requirements we used to filter these data. PPM associated variants had to be: i) heterozygous in Canino and Katy; ii) absent in the 10 self-incompatible cultivars analyzed, including Goldrich; and iii) shared by Canino and Katy (Fig. 3A). Only five SNV/SVs fulfilled all these conditions. Three of these variants were located in intergenic regions of the a*M*-supercontig, namely position/variants 247165/T>A, 234601_5/insATATAA, and 273855_68/del(AG)_7_). In addition, we detected an insertion in the seventh intron of *ParM-15*, 271210_21/ins(TAAA)_3_, that does not alter splicing sites. Lastly, an insertion of undetermined length was detected in exon three of *ParM-7* at 214579 (Fig. 3B).

Sequencing of the wild-type and mutant *ParM-7* alleles showed that the insertion present in the mutant allele was 358 bp in length. The wild-type allele (*M*) of *ParM-7* was amplified and sequenced from genomic DNA (gDNA) and pollen cDNA of CC-77, a tree with genotype *MM* obtained by selfing Canino. Mutant alleles (*m*) were sequenced from CC-67, a sibling of CC-77 with genotype *mm*, and K06-17, a tree derived by selfing Katy with genotype *mm* (Fig. 3C, Supplementary Fig. S2 and Supplementary Table S4). The mutant alleles from Canino and Katy were identical to each other and both were identical to the *M*-allele sequence, except for a 358 bp insertion in the third exon at position 332 of the coding region. The insertion disrupts the ORF by substituting a TTT phenylalaninecodon with a TGA stop codon, truncating the predicted protein at amino acid 111 (Fig. 3C). The 358 bp insertion is identical in size and similar in sequence, with 86.3% identity, to an insertion identified in the *SFB*_C_ gene by Vilanova *et al.* (2006) that was classified as a MITE type and named *Falling Stones* (*FaSt*) by Halász *et al.* (2014). The *ParM-7 m*-allele insertion also has the structural features of a *FaSt* MITE: 9 bp long target site duplications, high AT content at 75.7%, and flanking terminal inverted repeats (Fig. 3C).

Fig. 3D shows *ParM-7* genotyping in recombinants from G×C and K×K populations. A ~1.3 kb PCR product corresponding to the *m*-allele was obtained from all self-compatible individuals carrying the PPM (*Mm*/*mm*), including cultivars Portici and Corbató previously shown to carry the *m*-haplotype (Muñoz-Sanz *et al.*, 2017). This confirmed that the 358 bp insertion is linked in coupling with *m* in both populations. The insertion was not detected in self-incompatible recombinants and cultivars, all these showed a unique ~0.9 kb PCR-fragment (*MM*).

### *ParM-7* encodes a disulfide bond A-like oxidoreductase

BLASTP against the NCBI nr protein database showed that the predicted ParM-7 protein is similar to oxidoreductases containing a disulfide bond A-like (DsbA-like) domain (PF01323, *E*-value=3.34*E*^−26^; IPR001853). DsbA-like proteins contain the thioredoxin fold (IPR012336) and are members of the large thioredoxin (Trx)-like superfamily (Supplementary Table S6). Accordingly, we named *ParM-7 P. armeniaca M*-locus DsbA-like oxidoreductase, *ParMDO*. Trx fold proteins have a characteristic CxxC motif and modulate the redox state of target proteins. ParMDO displays the CPWC variant of this motif at position 19 (Cys19-PW-Cys22) (Fig. 3C).

Proteins similar to ParMDO are widespread in dicots. BLASTP of dicot sequences identified 185 proteins with *E*-values<1*E*^−80^ and similarity >55%, with usually no more than two hits per species. Proteins from Rosaceae, Solanaceae, and Brassicaceae were selected for comparison with ParMDO: *Prunus persica*, *P. mume* (Japanese apricot), *Malus×domestica* (apple), *Fragaria vesca* (strawberry), *Solanum lycopersicum* (tomato), *Nicotiana spp.* (*S*-RNase based gametophytic SI type), and *A. thaliana* (sporophytic SI, as an outgroup) (Supplementary Table S6). Pm015400 and ppa017665m from *P. mume* and *P. persica*, respectively, display the greatest similarity to ParMDO with *E*-values<1*E*^−150^ and likely represent ParMDO orthologs. Interestingly, the third highest scoring protein (1*E*^−99^), ppa011285m, is 99% similar to apricot ParM-8 and its encoding gene is adjacent to *ppa017665* (Fig. 1, Supplementary Tables S3 and S6).

**Fig. 4.**
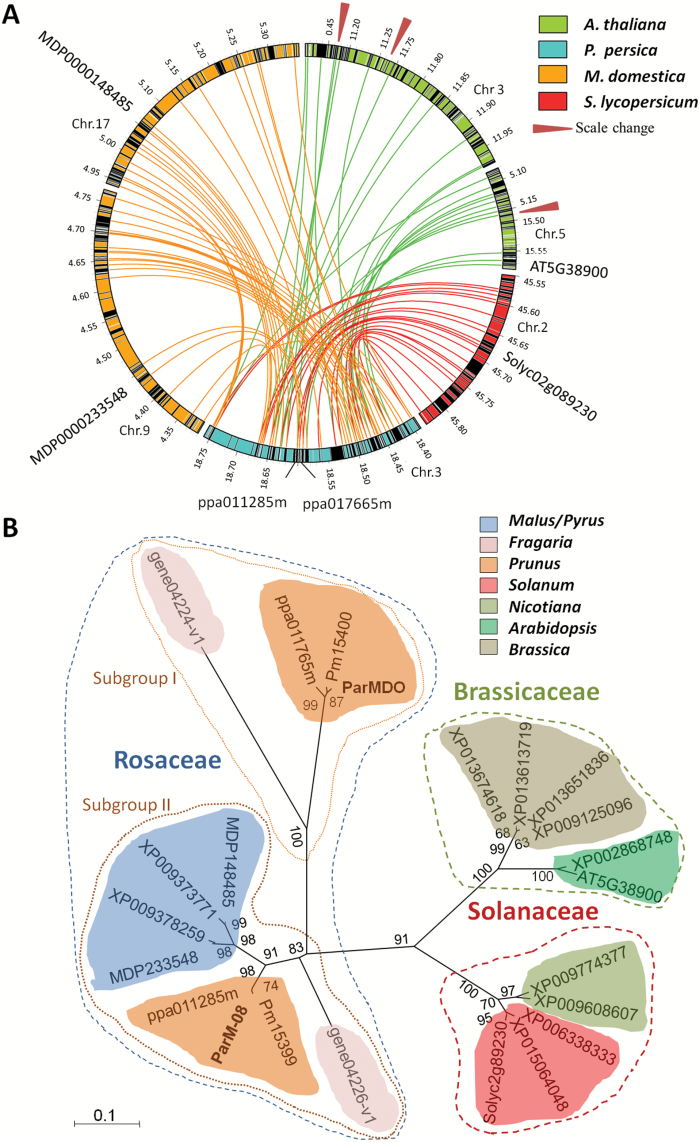
Ortholog analysis of *ParMDO*. (A) *M*-locus syntenic blocks between *P. persica*, *M. domestica*, *S. lycopersicum*, and *A. thaliana*. Black rectangles within circular genome regions represent gene annotation in scale. Red triangles indicate a scale change. Putative *ParMDO* and *ParM-8* orthologs are shown. (B) Clustering analysis of plant DsbA-like proteins. Three main clusters group DsbA-like proteins from Rosaceae (blue dashed line), Solanaceae (red), and Brassicaceae (green). The Rosaceae cluster is subdivided into subgroups I (ParMDO, their *Prunus* orthologs, and the *Fragaria* gene04224v1.0 protein) and II (all the rest including ParM-8). Bootstrap values>50% with 1000 replications are shown on the branches. The scale bar indicates the number of amino acid substitutions per site.

To identify putative orthologs to *ParMDO* and *ParM-8* in apple (Velasco *et al.*, 2010), strawberry (Shulaev *et al.*, 2011), tomato (Consortium TG, 2012), and Arabidopsis (Arabidopsis Genome Initiative, 2000) we followed a three step approach: 1) RBHanalysis; 2) identification of syntenic blocks; 3) phylogenetic inference based on clustering. RBHs for the peach protein ppa011285m, a putative ortholog of apricot ParM-8, were detected in all species examined. Moreover, all these, except for *Nicotiana*, were in turn RBH for the rest in their respective species. In striking contrast, no RBH was found for ppa017665m, a putative ortholog of ParMDO (Supplementary Table S7). RBH analysis was also used to identify syntenic blocks. A high degree of synteny was found between the ~0.4 Mb *M*-locus genomic region in peach, containing 62 putative genes, and equivalent sized *M. domestica* regions on homeologous chromosomes 9 and 17; 40 out of the 62 predicted proteins have a RBH in these two regions. *S. lycopersicum* chromosome 2 also contains an ~0.3 Mb region with significant synteny; 24 out of 62 proteins have RBHs. However, in spite of its phylogenetic proximity to *Prunus*, syntenic blocks in *A. thaliana* are distributed across four regions in chromosomes 3 and 5 (Fig. 4A and Supplementary Table S8). Fig. 4B shows that DsbA-like proteins from Brassicaceae, Solanaceae ,and Rosaceae clustered separately. Rosaceae proteins formed two sub-clusters. The first included ParMDO and their orthologs in *Prunus* ppa017665m and Pm15400. The second and largest included *Prunus* ppa011285m and ParM-8, *Malus* and *Pyrus* (Pyreae) proteins, and the *Fragaria* gene04226-v1.0-hybrid protein. The *Fragaria* gene04224-v1.0-hybrid protein branched separately from both sub-clusters but slightly closer to the first and, remarkably, displays a SPWC motif different from the typical CPWC catalytic motif present in all analyzed DsbA-like proteins (Supplementary Fig. S3).

## Discussion

### A *FaSt* MITE insertion in *ParMDO* (*ParM-7*) causes SC in Canino and Katy

PPM *m* causing SC in Canino and Katy was previously mapped to regions with an overlap of ~265 kb at the distal end of chromosome 3, the *M*-locus, which contains 42 putative genes (Zuriaga *et al.*, 2012; Zuriaga *et al.*, 2013). Here, high-resolution physical mapping localized the *M*-locus to ~134 kb and 16 genes were annotated. As prospects for obtaining further recombinants were not good due to the inherent limitations of woody species, we used expression data and variant calling to identify *m* candidates. *ParM-7* and *-14* emerged as the best candidates from expression data, as their expression patterns were similar to *SFB*, which determines *S*-specificity in pollen (Ushijima *et al.*, 2003; Vilanova *et al.*, 2006). Among all SNP and insertion deletion variants detected from 12 apricot cultivars in the ~134 kb *M*-locus, only the 358 bp *FaSt* MITE insertion in *ParM-7* fits all the criteria imposed by the known genetic behavior of PPM-based SC in Canino and Katy. *FaSt* MITE elements are thought to have arisen recently in subfamily *Prunoideae* where they accumulate in gene-rich regions of the genome (Halász *et al.*, 2014). Indeed, a very similar insertion also causing SC was previously identified in the Canino *SFB*_C_ allele (Vilanova *et al.*, 2006).

Furthermore, all other evidence is consistent with identifying the *FaSt* MITE insertion as the non-*S*-locus PPM conferring SC. First, it is linked in coupling with *m* in Canino and Katy and physically close (≤16.6 kb) to the microsatellite markers AGS.20, PGS3.23, and PGS3.62 that are closely linked to *m* (Zuriaga *et al.*, 2012; Zuriaga *et al.*, 2013). Second, all cultivars carrying the *m*-haplotype that have been tested are self-compatible (Muñoz-Sanz *et al.*, 2017). Third, sequence analysis shows that the insertion disrupts the *ParM-7 m*-allele ORF, so the predicted protein lacks four of the six exons. Fourth, *ParM-7* is preferentially expressed in pollen, consistent with a pollen function in SI. Based on this evidence, we conclude that the *FaSt* MITE insertion in *ParM-7* caused SC in the lineage leading to Canino and Katy.

### Pollen part SI function in *Prunus* is dependent on the candidate gene *ParMDO*

Sequence analyses identified *ParM-7* as a DsbA-like gene, *P. armeniaca M*-locus DsbA-like oxidoreductase, *ParMDO.* The predicted ParMDO protein is a member of the large and diverse protein Trx fold superfamily (Atkinson and Babbitt, 2009) characterized by a CxxC active site motif that confers thiol-disulfide redox activity important for folding, stability, and function of client proteins (Atkinson and Babbitt, 2009). Trx superfamily members have been associated with a wide range of sexual plant reproduction events from gametophyte formation to seed set (Traverso *et al.*, 2013). For example, Trx-h proteins, THL-1 and THL-2, inhibit S-locus receptor kinase (SRK) autophosphorylation during self-pollen rejection in the *Brassica* SI (Cabrillac *et al.*, 2001). Another Trx-h, NaTrxh, is localized in the extracellular matrix of the *Nicotiana* stylar transmitting tract and reduces S-RNases *in vitro* (Juárez-Díaz *et al.*, 2006). However, while Trx-h proteins typically reduce disulfide-bonds in clients, DsbA-like proteins are typically oxidases (Atkinson and Babbitt, 2009). For example, *Escherichia coli* DsbA-like proteins are thought to assist folding of periplasmic proteins such as RNaseI by catalyzing disulfide formation (Messens *et al.*, 2007). Although DsbA-like proteins appear to be widespread in plants, little is known about their functions and ParMDO may be the first one associated with a specific genetic function.

At this point it is premature to speculate deeply on the biochemical function of ParMDO but some kind of interaction with *S*-specificity determinants is expected. For example, ParMDO might maintain self-*S*-RNAses in active conformation, against the otherwise reducing environment of the pollen tube cytoplasm, facilitating self-pollen rejection. Alternatively, ParMDO could contribute to pollen-side SI function by oxidizing and inhibiting the hypothetical general inhibitor proposed by Matsumoto and Tao (2016). Biochemical experiments to identify ParMDO interacting proteins should help to define its function.

### The origin of ParMDO and SI in *Prunus*

The origin of the *S*-RNAse-based SI system in *Prunus* is uncertain because genetic and molecular features are substantially different from those displayed by other species, even rosaceous species such as apple and pear (Tao and Iezzoni, 2010). Since *ParMDO* is essential for SI in *Prunus*, the evolutionary history of this gene can provide new insights. *ParMDO* (*ParM-7*) and *ParM-8* are adjacent and highly homologous but *ParM-8* orthologs show a wide phylogenetic distribution unlike *ParMDO* (Fig. 4 and Supplementary Table S7). We suggest that a *ParM-8*-like gene was duplicated in a *Prunus* ancestor leaving *ParMDO* and *ParM-8* paralogs. *ParMDO* then gained preferential expression in pollen and adapted to function in SI, while *ParM-8* retained widespread expression and, probably, a more general function, reflecting a process of subfunctionalization (Force *et al.*, 1999).

Sequence analyses clearly show orthologs for *ParMDO* and *ParM-8* in all *Prunus* species examined but it is noteworthy that *ParMDO* has no orthologs in the tribe *Pyreae*, namely apple and pear. This could be explained by loss of a *ParMDO*-like gene in *Pyreae* or by a duplication that occurred only in the lineage leading to *Prunus*. In addition, while *ParM-8* has a clear ortholog in the more distant species *Fragaria vesca* (gene04226-v1.0), sequence homology does not fully support that *ParMDO* is the ortholog of gene04224-v1.0. However, genes encoding these two proteins in *Fragaria* are adjacent in the genome, similar to *ParMDO* and *ParM-8* in apricot. Thus, we suggest that gene04224-v1.0 and *ParMDO* may be orthologs and the lesser sequence identity can be attributed to subfunctionalization along the *Prunus* lineage. Accordingly, we tentatively favor that the proposed duplication of *ParM-8* occurred in a common ancestor of *Fragaria*, *Pyreae*, and *Prunus* before the subfamily split in Rosaceae ~62 million years ago. These observations may be consistent with the convergent evolution of the SI system proposed by Aguiar *et al.* (2015) where *Prunus* and *Fragaria* shared a common ancestral SI locus in Rosaceae, *S-RNase* and *SFB* gene lineages, while *Malus* evolved independently. The ambiguity in the exact moment of the *ParM-8* duplication and the fate of *ParMDO* could be resolved by more extensive analysis in Rosaceae.

In any case, identification of *ParMDO* as a strong candidate gene essential to pollen part SI function in *Prunus* opens new avenues to elucidate both the evolution and molecular mechanism of SI in this intriguing group.

## Supplementary data

Supplementary data are available at *JXB* online.

Protocol S1. NGS data pre-processing

Protocol S2. GAP closure in the a*M*-supercontig

Protocol S3. Unsupervised clustering analysis of gene expression

Fig. S1. Graphical maps of recombinants from G×C and K×K populations used to fine-mapping the *M*-locus.

Fig. S2. Complete nucleotide sequence of the *ParM-7* (*ParMDO*) gene obtained from Goldrich gDNA and cDNA.

Fig. S3. CLUSTALW alignment of plant DsbA-like proteins.

Table S1. Summary of Next Generation Sequencing (NGS) data.

Table S2. SSR and SNP markers developed for the fine-mapping of the *M*-locus.

Table S3. Apricot annotated genes within the ~134 kb high-resolution mapping region of the a*M*-supercontig.

Table S4. Primers used for RT-PCR analysis, *ParMDO* cDNA and gDNA synthesis and PCR-amplification of the *FaSt* MITE insertion.

Table S5. Basic and structural variant calling in WGS Illumina data from 12 apricot cultivars aligned to the a*M*-supercontig.

Table S6. BLASTP analysis of the predicted protein for *ParMDO*.

Table S7. RBH analysis of protein hits from the ParMDO BLASTP.

Table S8. RBH analyses to identify *M*-locus syntenic blocks between *P. persica* and *M. domestica*, *S. lycopersicum* and *A. thaliana*.

## Data deposition

Gene sequencing data. NCBI Genbank. Accession numbers KY499716, KY429940 and KY429941.

https://www.ncbi.nlm.nih.gov/nuccore/KY499716

https://www.ncbi.nlm.nih.gov/nuccore/KY429940

https://www.ncbi.nlm.nih.gov/nuccore/KY429941

## Supplementary Material

supplementary_figures_S1_S3_Tables_S1_S7

supplementary_Tables_S5

supplementary_Tables_S8

## Abbreviations:

DsbA-like: disulfide bond A-like
FaSt: Falling Stones
MITE: miniature inverted-repeat transposable element
NGS: next generation sequencing
PPMs: pollen-part mutations
RBH: reciprocal best hit
SC: self-compatibility
SFB: *S*-locus F-box genes
SI: self-incompatibility
SNPs: single nucleotide polymorphisms
SNVs: single nucleotide variants
SSRs: simple sequence repeats
SVs: structural variants
Trx: thioredoxin
WGS: whole genome sequencing.
