# Supplementary material for: A disulfide bond A-like oxidoreductase is a strong candidate gene for self-incompatibility in apricot (Prunus armeniaca) pollen
Source: J Exp Bot. 2017 Sep 25;68(18):5069–78. doi: 10.1093/jxb/erx336 (PMC5853662; doi:10.1093/jxb/erx336)
Supplement: supplementary_figures_S1_S3_Tables_S1_S7 [file erx336_suppl_supplementary_figures_s1_s3_tables_s1_s7.pdf]

Supplementary Data

Supplementary Protocol S1

NGS data pre-processing

Raw 454 data from BAC clones were filtered and trimmed (‘quality limit’=0.05 ‘ambiguous limit’=3 and ‘minimum length’=50 bp) by CLC Genomics Workbench 8.0.1. Trimmed sequences were aligned against pBeloBAC11 to remove cloning vector sequences. WGS raw Illumina data were also filtered and trimmed (‘quality limit’=0.05, ‘ambiguous limit’=2 and ‘minimum length’=20 bp) by CLC Genomics Workbench 8.0.1. Raw Illumina RNA-Seq data were trimmed (‘minimum quality score’=25 and ‘minimum length’=40) using FASTX-toolkit (http://hannonlab.cshl.edu/fastx_toolkit). Read quality was checked using FastQC v.0.10.1 (http://www.bioinformatics.babraham.ac.uk/projects/fastqc/) software. All WGS raw reads used for variant calling (including SRA downloaded sequences) were processed using the ‘run_trimmomatic_qual_trimming.pl’ script from the Trinity software to remove low-quality regions as well as vector and adaptor contaminants.

Supplementary Protocol S2

GAP closure in the a*M*-supercontig

Contigs from overlapping BAC clones that could not be joined by *GAP4* were assigned to different *M*-haplotypes by SSR genotyping, using markers evenly distributed in the *M*-locus (PGS3.71, PGS3.47, PGS3.23, PGS3.62, PGS3.63, PGS3.96, AGS.6 and 160J21-2). PCR conditions were the same described for SSR amplification in the ‘Nucleic acids extraction and Genotyping’ subsection. GAP closure was carried out as follows: i) BLASTN (Altschul *et al*., 1990) analysis (cutoff e-value $<10^{-3}$) using PGS3-SSR primer pair sequences against contig sequences to identify matching GAPs between end-sequences of adjacent contigs; ii) Unsolved GAPs were PCR-amplified with specific primers designed by Primer3 (Untergasser et al., 2012) from end-sequences of adjacent contigs. PCR conditions and purification procedure of PCR products were the same reported in the ‘Nucleic acids extraction and Genotyping’ subsection. Sanger sequencing conducted by Sistemas Genomicos S.L. (Paterna, Valencia, Spain). Resulting sequences were assembled through *Staden package*.

Supplementary Protocol S3

Unsupervised clustering analysis of gene expression

To filter out transcript artifacts and lowly expressed transcripts, only those with a value of counts per million (cpm)>1, at least in all replicates of one condition, were maintained. A between-sample normalization was made considering the total number of reads per library. Relationship between samples and replicates (technical and biological) was inspected by a multidimensional scaling (MDS) plot.

Supplementary Figures

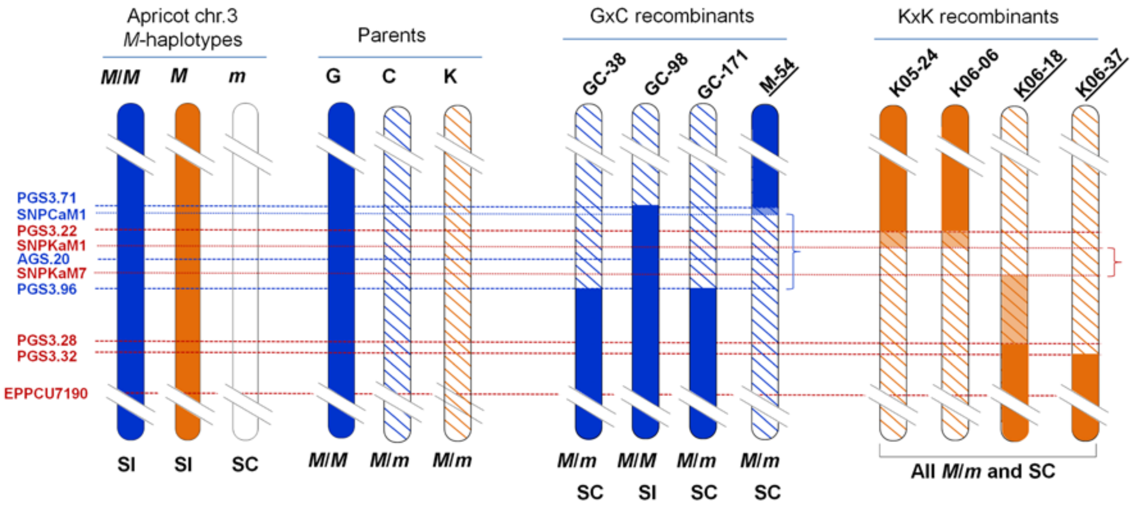

Supplementary Fig. S1. Graphical maps of recombinants from G×C and K×K populations used to fine-mapping the *M*-locus. *Vertical bars* represent the *M*-locus region in the apricot chr. 3. *Blue* and *orange* colors identify the *M*-haplotype in Goldrich/Canino and Katy, respectively, and *white* the mutated *m*-haplotype. *Blue/white* and *orange/white stripped bars* symbolize the *Mm* genotype. *M*-locus genotypes for parents Goldrich (G), Canino (C) and Katy (K), and recombinants are indicated at the bottom. Molecular markers delimiting the *M*-locus in Canino and Katy are written in *blue* and *orange*, respectively. Accordingly, recombinant breakpoints are shown by *horizontal blue* and *orange dashed* (for SSRs) or *dotted lines* (for SNPs), respectively. New recombinants incorporated in this work are *underlined*. *Transparent shaded* regions show approximately (not to scale) the *M*-locus size reduction after fine-mapping.

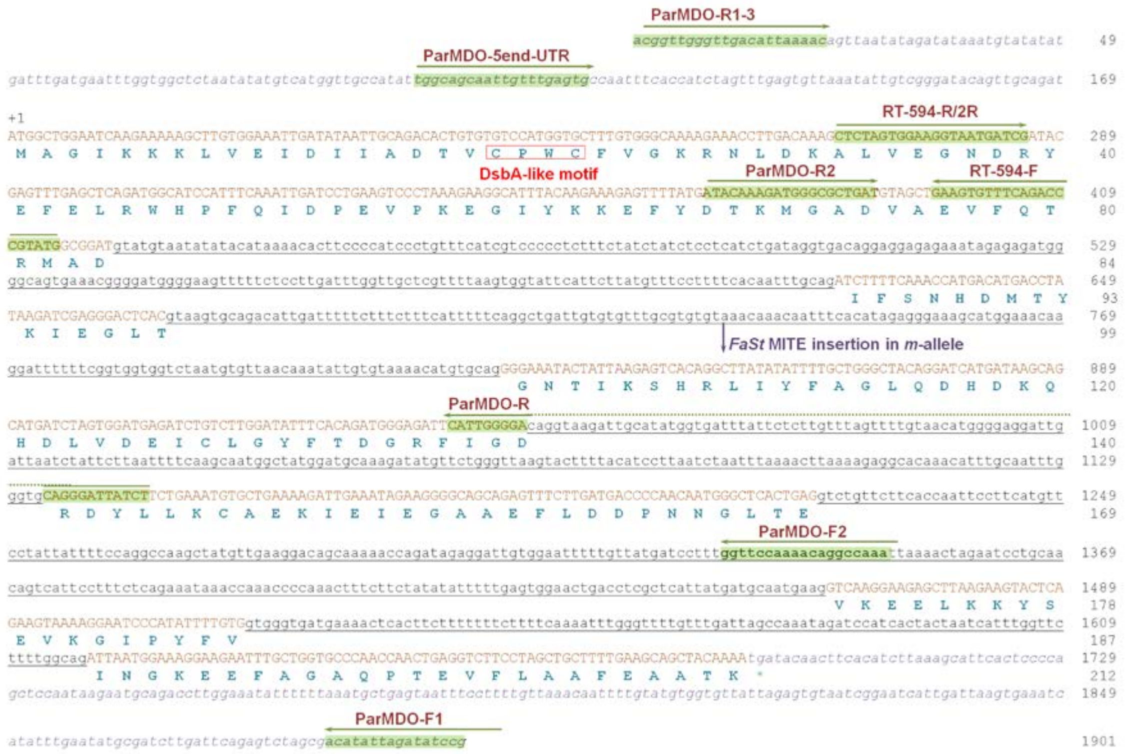

Supplementary Fig. S2. Complete nucleotide sequence of the *ParM-7* (*ParMDO*) gene obtained from Goldrich gDNA and cDNA. Exons (*red capital letters*), introns (*black lowercase*), 5'- and 3'-UTR regions (*black italic lowercase*) and the predicted aminoacid sequence (*green capital letters*), are indicated. Positions for primers used in RT-PCR analysis and cDNA synthesis (see Supplementary Table S4), DsbA-like motif and the *FaSt* MITE insertion are also shown.

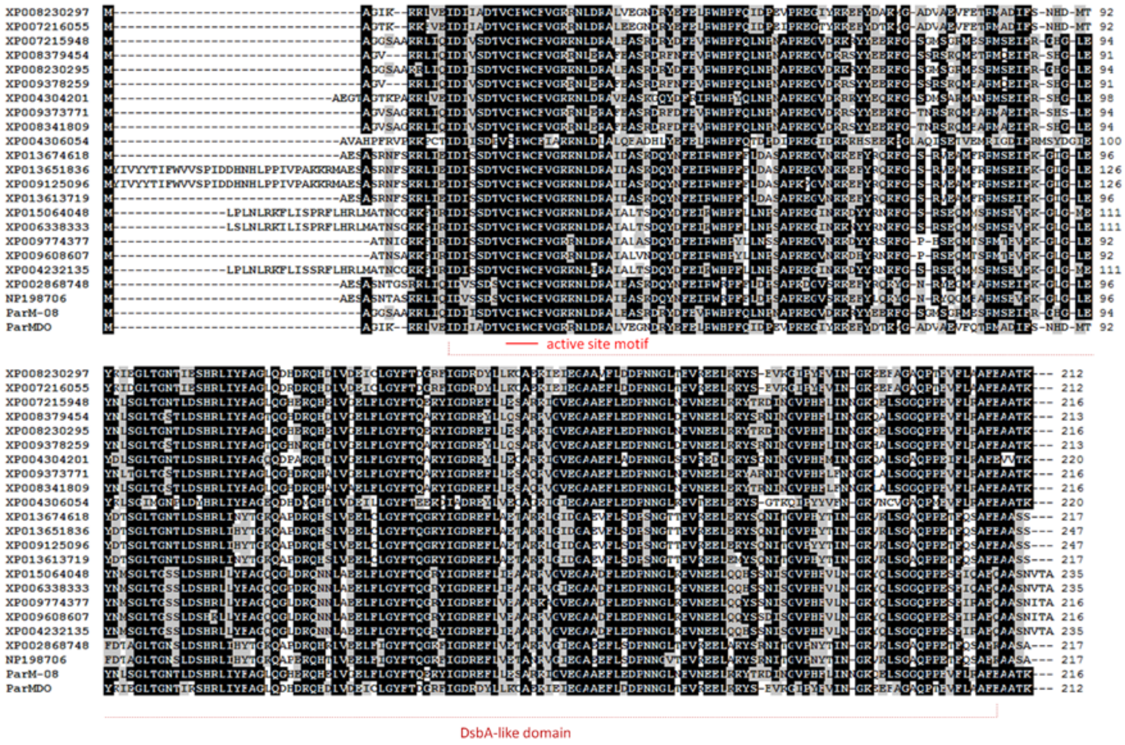

Supplementary Fig. S3. CLUSTALW alignment of plant DsbA-like proteins. The DsbA-like domain and the conserved CPWC active site motif are shown. Identical residues are highlighted in *black boxes* and *dashes* indicate gaps. Alternative protein IDs, when available, are listed in Supplementary Table S6 and Supplementary Table S7.

Supplementary Table S1. Summary of Next Generation Sequencing (NGS) data. NGS platform, DNA/RNA source, Sample (cultivar), Phenotype (SI/SC), Tissue (leaves, anthers or styles), Number and Average size (bp) of raw and cleaned sequences are indicated.

NGS	DNA/RNA source	Sample	Phe.	Tissue	Raw data Nº seqs.	Avg. size	Cleaned data Nº seqs.	Avg. size	Reference
454	BAC-215E14	Goldrich	SI[a]	-	29334	399.8	21909	377.9	This work
454	BAC-209M03	Goldrich	SI[a]	-	39135	408.9	28137	387.9	This work
454	BAC-108J24	Goldrich	SI[a]	-	27174	393.9	20890	370.4	This work
454	BAC-224A3	Goldrich	SI[a]	-	46679	402.9	34777	385.5	This work
454	BAC-234O11	Goldrich	SI[a]	-	16768	384.8	13094	363.8	This work
454	BAC-148M17	Goldrich	SI[a]	-	24553	299.4	23738	296.9	This work
454	BAC-253J12	Goldrich	SI[a]	-	9262	287.6	8969	284.8	This work
454	BAC-251L05	Goldrich	SI[a]	-	16348	292.1	15718	288.9	This work
454	BAC-160J21	Goldrich	SI[a]	-	19125	306.3	18481	301.7	This work
454	BAC-95D02	Goldrich	SI[a]	-	9374	299.3	9065	299.2	This work
454	BAC-159P08	Goldrich	SI[a]	-	9937	295.5	9676	291.6	This work
454	BAC-161F24	Goldrich	SI[a]	-	10233	293.9	9931	291.6	This work
Illumina	gDNA	Canino	SC[a]	Lea.	373801518	101	371672380 (129438652)[e]	99.3 (99.6)	This work
Illumina	gDNA	Katy	SC[b]	Lea.	69669448	101	69042494	98.8	This work
Illumina	gDNA	Goldrich	SI[a]	Lea.	137954275	101	136391075	92.7	C. Dardick[f]
Illumina	gDNA	SEO	SI[a]	Lea.	156657196	101	149154961	94.9	C. Dardick[f]
Illumina	gDNA	Orange Red	SI[a]	Lea.	38381772	101	35912056	94.2	SRA[g]
Illumina	gDNA	Stella	SI[a]	Lea.	276885698	101	273836121	96.7	SRA[g]
Illumina	gDNA	Lambertin	SI[a]	Lea.	275826032	101	271308860	94.7	SRA[g]
Illumina	gDNA	Veecot	SI[a]	Lea.	72451224	101	71965887	97.6	SRA[g]
Illumina	gDNA	Harcot	SI[a]	Lea.	218574844	101	213377770	96.1	SRA[g]
Illumina	gDNA	Perfection	SI[a]	Lea.	167226720	101	165422097	96.7	SRA[g]
Illumina	gDNA	Moniquí	SI[a]	Lea.	104169396	101	102432453	93.6	SRA[g]
Illumina	gDNA	Velázquez	SI[a]	Lea.	46142574	101	45802173	97.9	SRA[g]
Illumina	RNA	Goldrich	SI[a]	Ant.[c]	122397834	107	122338874	106.5	This work
Illumina	RNA	Goldrich	SI[a]	Sty.[d]	122313850	107	122268676	106.5	This work
Illumina	RNA	Goldrich	SI[a]	Lea.[d]	135741242	107	135688624	106.6	This work
Illumina	RNA	Canino	SC[a]	Ant.[c]	159854696	107	159774857	106.5	This work
Illumina	RNA	Canino	SC[a]	Sty.[d]	123966784	107	124313835	106.3	This work
Illumina	RNA	Canino	SC[a]	Lea.[d]	110887662	107	110843339	106.6	This work
Illumina	RNA	Katy	SC[b]	Ant.[c]	130685722	107	130624896	106.5	This work
Illumina	RNA	Katy	SC[b]	Lea.[d]	103966200	107	103922997	106.6	This work

[a] Burgos L, Alburquerque N, Egea J. 2004. Review. Flower biology in apricot and its implications for breeding. *Span J Agric Res* 2:227-241.
[b] Russell D. 1998. *The stonefruit cultivar system (A database of worldwide stonefruit cultivars and rootstocks).* Queensland, Australia: Department of Primary Industries.
[c] 3 biological replicates / 2 technical replicates per biological replicate
[d] 2 biological replicates / 2 technical replicates per biological replicate
[e] Due to the high depth of coverage, 1/3 of cleaned sequences (between brackets) were randomly selected to be used in subsequent analysis.
[f] Sequences kindly provided by Chris Dardick (USDA)
[g] Sequences were downloaded from the NCBI Sequence Read Archive (SRA) repository (Mariette *et al*., 2016).

Supplementary Table S2. SSR and SNP markers developed for the fine-mapping of the *M*-locus. Marker positions in the a*M*-supercontig and primer sequences are indicated. Repeat motifs and allele sizes of SSRs (AGS and 160J21) and allelic composition of SNPs (SNPCaM and SNPKaM) in cultivars Goldrich, Canino and Katy are also shown.

**Name**	**F/R**	**Primer sequence**	**Repeat motif**	**Start on**	**Goldrich alleles**	**Canino alleles**	**Katy alleles**
SNPCaM1	F	TAATGTGAGTCTTGGACGTG	-	33687/**33688**	T **C**	G **C**	-
	R	CTGTCCTTTTTGGATTCCTGA			G **T**	G **T**	
AGS.3	F	AAAATGTTGGGCTCCCTTTC	(TTC)7	37610	164	164	164
	R	TGAACGACTTGGGGGAATAG					
AGS.4	F	TTGGCATCTCTGGTGCAAT	(AT)7	32804	438/470	470	441/470
	R	ACAATGAGGTTGCCTTCGTC					
AGS.6	F	GAGTGGCCGATACCTGTTCT	(AATT)4	70573	238/241	241	238/241
	R	AATGATGGGTTTTGGGTGTG					
AGS.7	F	TTCGGCTTCCAATCATAAGG	(TC)14	76026	N.A.[a]	N.A.	N.A.
	R	AGAAATGGAGGTGTCGTTGG					
AGS.8	F	TTCGTAGCATTCTGGGGTTT	(GA)10	102236	215/230	250	238/250
	R	GGGGGCTTGAATGATAGGAT					
AGS.9	F	AGGCATGTGTGTTTGACACC	(AAT)5	103440	225/226	225	225
	R	AATGTGGACATGAAGCACCA					
AGS.10	F	CTCCCATGGAAAACCTCAAA	(CT)30	115967	198/203	198	198/203
	R	GGGGCATTTCTGATGGTAAA					
AGS.11	F	TTTGCCTTCATACACCTAGCC	(AT)10	116715	261/269	261	261
	R	CACAAGCATGAGACCATCCA					
AGS.12	F	ACGATGAATTTGAAGACGATGA	(CT)9+(TTG)6	136201	193/210	193/210	193/210
	R	ACCTTCACTGCCAAATTCCCTATC					
SNPKaM1	F	CAAGCAAGGGGCAATTAACA	-	142155	-	-	A/G
	R	CGCTAACACCAGAGGAAACTG					
SNPKaM2	F	GGTGTTCATCAGAAGCAGCA	-	146316	-	-	G/A
	R	CATGTTCATTCAACGGCATA					
AGS.14	F	AGAAGGCCCTGCACCTAAAT	(CCT)6	152359	219/236	219/236	219/236
	R	CATAAACTCAGGGGCTTGGA					
SNPKaM3	F	ACGTCTCATTTCATCCCTGGT	-	153066	-	-	T/C
	R	GGCTGCAGAAAGAACATGAAG					
SNPKaM4	F	GCAAGAGGTCAACACCAAAAG	-	164682	-	-	A/C
	R	CTCAAAAGGCTGTTGCTCTGT					
SNPKaM5	F	TGCCGACTATCAACAGTAAACC	-	171071	-	-	G/A
	R	GACATGCATCTTCCTTGAGA					
AGS.17	F	AAAAACACCTCTCCCGACAA	(TA)6	177634	190/199	199	190/199
	R	AGCGGCGATACTCGTTTTAC					
AGS.18	F	CAATGGACGAGTAGGGGTGT	(AT)12	175974	387/389	389	387/389
	R	TTGGGTTTGGAGAGGTTTTG					
AGS.19	F	TATCATGCGTCGCTCTCAAG	(AT)10	208676	235/251	251	235/251
	R	CACAATTGGATGTCGAAACG					
AGS.20	F	CGAACGAGAGGGAAAAATGA	(AT)10	224961	188	188/190	190/192
	R	AACTGATTCCGAACCACAGG					
AGS.21	F	TGTGTCCCTCGATCCTTACC	(TA)10	236517	514/518	518	503/505
	R	CTATCCGATTTCCAATCCGACA					
AGS.22	F	AGTTCAAGCGGCTTTCAGAT	(TA)4+5	244325	171	171	171
	R	AATGCCAGTCCTTCGATGAG					
AGS.23	F	TACAATCAATGGCGGATTCA	(TA)8	250156	N.A.	N.A.	N.A.
	R	TTTCTTCGTCTGAGCCTTTGA					
AGS.24	F	TCCAAAAGAAGCAACGTCAA	(GA)23	250524	N.A.	N.A.	N.A.
	R	CCATGCTTGGGTTAAAGTGG					

AGS.26	F	AATATTGGTCCCCCTCCAAG	(GTT)4+5	252416	240/258	240/258	240/258
	R	GCAAGAGAAACGAAAAGCTCA					
SNPKaM6	F	AGCCACCATGCACCCTATAC	-	273675	-	-	A/G
	R	TCACATGGTAACCAAGCTCCT					
AGS.27	F	GTTGCACGGAAATTCCAGAT	(AG)14	275668	175/182	175	175/182
	R	GTGTGCGTCTGTGTGGGTAG					
SNPKaM7	F	CACGAGGGCCTCTATTTTGT	-	276184	-	-	T/C
	R	CTCCTTTTGGTGCATGTGTG					
AGS.28	F	GGGTCCTCAACAGACCAAAG	(GA)9	276953	179/182	179/182	N.A.
	R	AGGTGCACGTGGATAGACCT					
SNPKaM8	F	AATGTGTTTGGACAAGTCACG	-	277811	-	-	C/T
	R	CACACTTCACTCCAACCGAAT					
SNPKaM9	F	GGCTAATGTGCAAGAGGTTTG	-	285823	-	-	C/T
	R	GGGAGAGAAGTATGCAGAGCA					
SNPKaM10	F	CCCGTTTTGGAGAATAGAAGAC	-	295239	-	-	A/G
	R	CCTATGGAGATAGGTTCCTTGA					
AGS.29	F	ACGTCGTTTTGGCAATGTTT	(ATA)4	295820	N.A.	N.A.	N.A.
	R	ACATGTGCCCTTTGTTTGTG					
AGS.30	F	CCGCACGGCTATACTGTCTAA	(AT)13	309620	203	203	195/203
	R	ACAGGCTGGATGCTTTGTCT					
AGS.31	F	AATTGCCCCCGTCTATCAC	(CT)5+5	313614	194/196	196	194/196
	R	GAGAATGGGTGGGGTAGGAC					
AGS.32	F	CCCAGCTGAAATGGGAATAC	(AT)11	315102	282/297	282/297	282/297
	R	GCATGCATCATGTTTTCCTG					
AGS.33	F	CACCCCCTCCCTCTCTTTTA	(CT)10	319478	ML[b]	ML	ML
	R	CATGTTGGTCGATTTGTAGCC					
AGS.34	F	TCACCAGCTGACGTGGTAGT	(CT)16	320217	N.A.	N.A.	N.A.
	R	CAATTCCTCATCTGGGCAGT					
160J21-7	F	ACTTGAGATTGATGCTCCCATT	(AT)11	332558	164	164	164
	R	ACCAACAGCTCCAAATTAAACC					
AGS.35	F	CAGGCCTCAAAGGCAAAAC	(CTAGGCGGCT)21	335737	N.A.	N.A.	N.A.
	R	CACCCCCTCCCTCTCTTTTA					
160J21-6	F	CCTTCACCAACTTCAAACCCTA	(GA)6	336648	189	189/191	191/193
	R	TTGTTCCTATTTTCGATACCCG					
160J21-5	F	CTACTGCTGAACGACCAAAACA	(AT)11+(CA)6	337460	223/238	223/238	223/238
	R	AACGGATTTTCATGGTAGATGC					
160J21-4	F	CCTCTCTCACTCAACCTGCTCT	(TC)18	338507	N.A.	N.A.	N.A.
	R	AAGCGTTTAGCCAAGGAACTAA					
AGS.36	F	ACCCAGAGGTACCCTTCGAG	(CTAGGCGGCT)14	343974	ML	ML	ML
	R	ACTTCCATCACCCTTCGTCA					
160J21-3	F	TGTGAAGGTCATGGGTTTACAA	(GT)9	347903	N.A.	397/403	397/403
	R	ACGGTTTTCCAAGTACAACGTC					
160J21-2	F	GGTTGGACTGCTTTTCATTCTT	(TAA)16	349292	350/352	350/354	352/354
	R	ATTTCTTTGGAGTTGAGGTGGA					
AGS.37	F	TCAAATCTCTTGGGCCAATC	(GGT)6	350097	256/264	264	264/270
	R	ATTCACTACCCCCACAACCA					
AGS.38	F	CATCATGTACGGAAGCACCA	(AT)12	353246	ML	219/221	219/221
	R	CCGTTGGACATTCCTTTTTC					
AGS.39	F	CTCGCGAAACCCTAACATTT	(TC)9+9	338506	N.A.	N.A.	N.A.
	R	ACCGGGAGAAAACGACAGT					
AGS.40	F	CATCATGTACGGAAGCACCA	(AT)12	353246	N.A.	222	220/222
	R	CCGTTGGACATTCCTTTTTC					
AGS.41	F	ATGGAAGATGATTGCCCAAC	(AT)15	366351	ML	342	342/344
	R	TTGTCATGTTGATGCCCTGT					

[a] N.A. not amplified; [b] M.L. multi-locus pattern

Supplementary Table S3. Apricot annotated genes within the ~134 kb high-resolution mapping region of the a*M*-supercontig. Start/end positions within the a*M*-supercontig, gene and protein sizes and homology rates for CDS and predicted proteins with putative orthologues in peach v1.0 and v2.0 (http://www.rosaceae.org) and *P. mume* (BioProject PRJNA171605) are indicated**.**

**P.armeniaca** annotated genes	Start position	End position	Size gene (nt)/ protein (aa)	Putative orthologue in **P. persica v1.0**	Homology rate: CDS/protein	Putative orthologue in **P. persica v2.0**	Homology rate: CDS/protein	Putative orthologue in **P.mume**	Homology rate: CDS/protein
ParM-16[a]	141885	144673	2789/420	ppa004594m	82.40/81.02	Prupe.3G248200.3	98.57/97.85	Pm015411	83.10/82.80
ParM-1	148466	150367	1902/182	ppa012139m	99.27/100	Prupe.3G248300.1	99.27/100	Pm015410	99.27/99.45
---	---	---	---	---	---	---	---	Pm015409	---
ParM-2	150485	175756	25272/4966	ppa000002m	98.53/98.45	Prupe.3G248400.1	98.53/98.45	Pm015408	99.33/98.98
---	---	---	---	ppa026731m	---	---	---	---	---
---	---	---	---	---	---	---	---	Pm015407	---
ParM-17[b]	179584	180691	1108/---	ppa023507m	---	Prupe.3G248500.1	---	Pm015406	---
ParM-3	183473	185099	1627/424	ppa005351m	96.81/94.02	Prupe.3G248600.1	96.81/94.02	Pm015405	99.60/98.57
ParM-4	192717	194311	1595/360	ppa011450m	47.37/32.76[c]	Prupe.3G248700.1	93.70/94.82	Pm015403	98.15/98.61
ParM-5	199905	206573	6669/786	ppa001620m	95.08/99.24	Prupe.3G248800.1	95.08/99.24	Pm015402	93.30/98.98
ParM-6	209304	213664	4361/227	ppa011007m	98.68/99.12	Prupe.3G248900.1	98.68/99.12	Pm015401	100/100
ParM-7	213665	215507	1843/212	ppa017665m	97.34/96.23	Prupe.3G249000.1	97.34/96.23	Pm015400	99.06/98.58
ParM-8	215917	218605	2689/216	ppa011285m	98.92/99.54	Prupe.3G249100.1	98.92/99.54	Pm015399	99.08/99.54
ParM-9	219162	223345	4184/477	ppa005069m	99.09/99.16	Prupe.3G249200.1	99.09/99.16	Pm015398	99.37/98.95
ParM-10	223346	228009	4664/269	ppa010249m	98.05/95.33	Prupe.3G249300.1	97.04/85.88	Pm015397	98.77/97.03
---	---	---	---	---	---	---	---	Pm015396	---
---	---	---	---	---	---	---	---	Pm015395	---
ParM-11	238908	243677	4770/245	ppa010548m	99.05/98.78	Prupe.3G249400.1	99.05/98.78	Pm015394	99.73/99.18
ParM-12	248132	249452	1321/112	ppa026503m	98.82/99.11	Prupe.3G249500.1	98.82/99.11	Pm015393	99.12/100
ParM-18[b]	250829	253047	2219/---	ppa016385m	---	Prupe.3G249600.1	---	Pm015392	---
ParM-19[b]	258899	260109	1211/---	ppa1027219m	---	Prupe.3G249700.1	---	Pm015391	---
ParM-13	261087	266468	5382/579	ppa003386m	98.79/99.31	Prupe.3G249800.1	98.79/99.31	Pm015390	99.77/99.65
ParM-14	266578	269011	2434/356	ppa007756m	99.07/99.44	Prupe.3G249900.1	99.07/99.44	Pm015389	99.72/99.16
ParM-15	270633	273254	2622/464	ppa005994m	99.08/99.07	Prupe.3G250000.1	99.20/99.28	Pm015388	99.64/99.78

[a] Only partial sequence since the ~134 kb region starts on the left flanking marker SNPKaM1 at position 142155

[b] Rough annotation by blasting putative orthologues in peach v1.0 (http://www.rosaceae.org) against the a*M*-supercontig

[c] Low homology is due to wrong annotation in peach v1.0 (http://www.rosaceae.org)

Supplementary Table S4. Primers used for RT-PCR analysis, *ParMDO* cDNA and gDNA synthesis and PCR-amplification of the *FaSt* MITE insertion.

Gene	Primers	Sequence 5'-3'	Reference
ParM-6	ParM6-F	5'-AGAAGTGAAAGCTGCCCTGTT-3'	This work
	ParM6-R	5'-TCTTCAGCTGTTGTTGGCTTC-3'	This work
ParM-7 (*ParMDO*)	RT594-F	5'-CATACGGGTCTCAAACACTTC-3'	This work
	RT594-R	5'-CTCTGGAGGAAGGTAATGATCG-3'	This work
ParM-8	ParM8-F	5'-CCATGGCCTGGAATATAACCT-3'	This work
	ParM8-R	5'-AATAGCTCACCCAGAAGCTCA-3'	This work
ParM-14	ParM14-F	5'-GCTTCTGCAACTCACCTTTTG-3'	This work
	ParM14-R	5'-CATCTTGGACGATGATGAGGT-3'	This work
S-RNase	Pru-C2	5'-CTTTGGCCAAGTAATTAT TCAAACC-3'	Tao et al. 1999[a]
	Pru-C4R	5'-GGATGTGGTACGATTGAAGCG-3'	Tao et al. 1999[a]
SFB	RT-SFB1-F	5´-GGCAGCTCGAGTTTTGTTAGCATAC-3´	Zuriaga et al. 2013
	RT-SFB1-R	5´-GGAACCCGAATTGGAGAGAAACGAG-3´	Zuriaga et al. 2013
actin	ACT3	5´-CTTCTTACTGAGGCACCCCTGAAT-3´	De la Fuente et al. 2015[b]
	ACT4	5´-AGCATAGAGGGAGAGAACTGCTTG-3´	De la Fuente et al. 2015[b]
sand-like	SANDF	5'-TCGTGGGTACCAGGAAAACGACAT-3'	Rios (p. comm..)
	SANDR	5'-CCTGCTAGCTTGTGTTCATCTCCA-3'	Rios (p. comm..)
ParMDO cDNA *5´-end*	ParMDO-R	5'-AGATAATCCCTGTCCCCAATG-3'	This work
	ParMDO-5end-UTR	5'-TGGCAGCAATTGTTTGAGTG-3'	This work
ParMDO cDNA *3´-end*	ParMDO-F1	5'-GTTCTCTTGCCGGATATCTAATATGT-3'	This work
	RT594-2R	5'-CTCTAGTGGAAGGTAATGATCG-3'	This work
FaSt MITE insertion	ParMDO-F2	5'-TTTGGCCTGTTTTGGAACC-3'	This work
	ParMDO-R2	5'-ATACAAAGATGGGCGCTGA-3'	This work
ParMDO gDNA	ParMDO-R1-3	5'-ACGGTTGGGTTGACATTAAAAC-3'	This work

[a] Tao R, Yamane H and Sugiura A. 1999. Molecular typing of S-alleles through identification, characterization and cDNA cloning for S-RNAses in sweet cherry. *J Am Soc Hort Sci* **124**:224-233

[b] De la Fuente L *et al*. 2015. Genome-wide changes in histone H3 lysine 27 trimethylation associated with bud dormancy release in peach. *Tree Genet. Genom.* **11:** 45.

Supplementary Table S6. BLASTP analysis of the predicted protein for *ParMDO*. 'GenBank accession number' column shows selected hits (see Materials and Methods) from the BLASTP against the nr protein database using ParMDO as query. 'Genome protein database code' refers to the annotated ID in the corresponding protein database. The species of origin for each accession is indicated in the 'NCBI description' column.

**GenBank accession number**	**Genome protein database code**	**Max score**	**Query coverage**	**E-value**	**Identity**	**NCBI description**
XP_008230297.1	Pm15400	429	100%	7.0E-154	98%	Uncharacterized protein LOC103329582 [*Prunus mume*]
XP_007216055.1	ppa017665m	421	100%	9.0E-151	96%	Hypothetical protein PRUPE_ppa017665mg [*Prunus persica*]
XP_007215948.1	ppa011285m	291	100%	1.0E-99	62%	Hypothetical protein PRUPE_ppa011285mg [*Prunus persica*]
XP_008379454.1	MDP0000233548	291	100%	3.0E-99	62%	Uncharacterized protein LOC103442449 [*Malus domestica*]
XP_008230295.1	Pm15399	291	100%	1.0E-99	62%	Uncharacterized protein LOC103329581 [*Prunus mume*]
XP_004304201.1	gene04226-v1.0-hybrid	284	99%	2.0E-96	60%	Uncharacterized protein LOC101310818 [*Fragaria vesca* subsp. vesca]
XP_008341809.1	MDP0000148485	283	100%	3.0E-96	59%	Uncharacterized protein LOC103404656 [*Malus domestica*]
XP_004306054.1	gene04224-v1.0-hybrid	262	98%	5.0E-88	62%	Uncharacterized protein LOC101292444 [*Fragaria vesca* subsp. vesca]
XP_009774377.1		256	98%	5.0E-85	53%	Uncharacterized protein LOC104224422 [*Nicotiana sylvestris*]
XP_009608607.1		256	97%	5.0E-85	54%	Uncharacterized protein LOC104102573 isoform X2 [*Nicotiana tomentosiformis*]
XP_009608606.1		256	97%	6.0E-85	54%	Uncharacterized protein LOC104102573 isoform X1 [*Nicotiana tomentosiformis*]
XP_004232135.1	Solyc02g089230.2.1	255	97%	6.0E-85	54%	Uncharacterized protein LOC101251049 [*Solanum lycopersicum*]
NP_198706.1	AT5G38900.1	251	97%	1.0E-87	55%	Thioredoxin superfamily protein [*Arabidopsis thaliana*]

Supplementary Table S7. RBH analysis of protein hits from the ParMDO BLASTP. An all (protein queries in *bold*) to all (genome-predicted peptides) pairwise BLASTP comparison between NCBI taxids (A) *P. persica*, *M. domestica*, *F.vesca*, (B) *S. lycopersicum*, *N. bethamiana* and *A. thaliana*. Only hits with *E*-values lower than 10^{-5} are shown (identity % is also indicated).

A

Specie		Prunus persica			Malus domestica			Fragaria vesca		
	Query	Hit	E-value	Ident	Hit	E-value	Ident	Hit	E-value	Ident
Prunus persica	**XP_007216055.1**	XP_007216055.1	1.E-156	100%	XP_008379454.1	4.E-98	62%	XP_004304201.1	9.E-98	61%
	(ppa017665m)	XP_007215948.1	3.E-99	62%	XP_008341809.1	3.E-95	59%	XP_004306054.1	3.E-87	63%
	(ParMDO)									
	**XP_007215948.1**	XP_007215948.1	9.E-161	100%	XP_008341809.1	8.E-141	87%	XP_004304201.1	9.E-131	80%
	(ppa011285m)	XP_007216055.1	3.E-99	62%	XP_008379454.1	7.E-139	86%	XP_004306054.1	4.E-85	60%
Malus domestica	**XP_008379454.1**	XP_007215948.1	3.E-139	86%	XP_008379454.1	1.E-159	100%	XP_004304201.1	4.E-128	80%
	(MDP0000233548)	XP_007216055.1	2.E-98	62%	XP_008341809.1	4.E-146	91%	XP_004306054.1	4.E-83	58%
	(MDP0000191902)									
	**XP_008341809.1**	XP_007215948.1	4.E-141	87%	XP_008341809.1	4.E-161	100%	XP_004304201.1	2.E-124	75%
	(MDP0000148485)	XP_007216055.1	2.E-95	59%	XP_008379454.1	4.E-146	91%	XP_004306054.1	1.E-83	58%
Fragaria vesca	**XP_004304201.1**	XP_007215948.1	8.E-131	80%	XP_008379454.1	7.E-128	80%	XP_004304201.1	3.E-166	100%
	(gene04226-v1.0-hybrid)	XP_007216055.1	8.E-98	61%	XP_008341809.1	4.E-124	75%	XP_004306054.1	2.E-82	58%
	**XP_004306054.1**	XP_007216055.1	3.E-87	63%	XP_008341809.1	2.E-83	58%	XP_004306054.1	9.E-165	100%
	(gene04224-v1.0-hybrid)	XP_007215948.1	4.E-85	60%	XP_008379454.1	8.E-83	58%	XP_004304201.1	2.E-82	58%
Solanum lycopersicum	**XP_004232135.1**	XP_007215948.1	8.E-109	68%	XP_008341809.1	5.E-110	67%	XP_004304201.1	5.E-101	63%
	(Solyc02g089230.2.1)	XP_007216055.1	5.E-85	54%	XP_008379454.1	1.E-108	69%	XP_004306054.1	6.E-72	51%
Nicotiana	**XP_009774377.1**	XP_007215948.1	3.E-108	70%	XP_008341809.1	6.E-109	70%	XP_004304201.1	2.E-101	65%
		XP_007216055.1	2.E-85	54%	XP_008379454.1	1.E-107	69%	XP_004306054.1	9.E-73	51%
	**XP_009608607.1**	XP_007215948.1	8.E-110	70%	XP_008341809.1	1.E-108	68%	XP_004304201.1	2.E-101	64%
		XP_007216055.1	4.E-86	54%	XP_008379454.1	6.E-108	69%	XP_004306054.1	4.E-74	51%
	**XP_009608606.1**	XP_007215948.1	1.E-109	70%	XP_008341809.1	3.E-108	68%	XP_004304201.1	3.E-101	63%
		XP_007216055.1	6.E-86	54%	XP_008379454.1	1.E-107	69%	XP_004306054.1	1.E-73	51%
Arabidopsis thaliana	**NP_198706.1**	XP_007215948.1	2.E-103	65%	XP_008341809.1	5.E-100	63%	XP_004304201.1	7.E-101	63%
	(AT5G38900.1)	XP_007216055.1	9.E-84	55%	XP_008379454.1	2.E-97	63%	XP_004306054.1	1.E-68	49%

B

Specie		*Solanum lycopersicum*			*Nicotiana*			*Arabidopsis thaliana*		
	Query	Hit	E-value	Ident	Hit	E-value	Ident	Hit	E-value	Ident
Prunus persica	**XP_007216055.1**	XP_004232135.1	6.E-85	54%	XP_009608607.1	1.E-85	54%	NP_198706.1	2.E-83	55%
	(ppa017665m)				XP_009608606.1	2.E-85	54%			
	(ParMDO)				XP_009774377.1	6.E-85	54%			
	**XP_007215948.1**	XP_004232135.1	1.E-108	68%	XP_009608607.1	2.E-109	70%	NP_198706.1	5.E-103	65%
	(ppa011285m)				XP_009608606.1	4.E-109	70%			
					XP_009774377.1	8.E-108	70%			
Malus domestica	**XP_008379454.1**	XP_004232135.1	7.E-109	69%	XP_009608607.1	1.E-107	69%	NP_198706.1	3.E-97	63%
	(MDP0000233548)				XP_009608606.1	2.E-107	69%			
	(MDP0000191902)				XP_009774377.1	2.E-107	69%			
	**XP_008341809.1**	XP_004232135.1	3.E-110	67%	XP_009774377.1	9.E-109	70%	NP_198706.1	6.E-100	63%
	(MDP0000148485)				XP_009608607.1	2.E-108	68%			
					XP_009608606.1	4.E-108	68%			
Fragaria vesca	**XP_004304201.1**	XP_004232135.1	5.E-101	63%	XP_009608607.1	5.E-101	64%	NP_198706.1	1.E-100	63%
	(gene04226-v1.0-hybrid)				XP_009774377.1	7.E-101	65%			
					XP_009608606.1	8.E-101	63%			
	**XP_004306054.1**	XP_004232135.1	7.E-72	51%	XP_009608607.1	1.E-73	51%	NP_198706.1	3.E-68	49%
	(gene04224-v1.0-hybrid)				XP_009608606.1	3.E-73	51%			
					XP_009774377.1	3.E-72	51%			
**Solanum lycopersicum**	**XP_004232135.1**	XP_004232135.1	4.E-177	100%	XP_009608606.1	3.E-157	89%	NP_198706.1	1.E-104	63%
	(Solyc02g089230.2.1)				XP_009608607.1	5.E-150	91%			
					XP_009774377.1	6.E-150	92%			
Nicotiana	**XP_009774377.1**	XP_004232135.1	2.E-150	92%	XP_009774377.1	7.E-162	100%	NP_198706.1	9.E-103	65%
					XP_009608606.1	1.E-153	95%			
					XP_009608607.1	1.E-153	95%			
	**XP_009608607.1**	XP_004232135.1	2.E-150	91%	XP_009608607.1	5.E-162	100%	NP_198706.1	7.E-104	65%
					XP_009608606.1	7.E-162	100%			
					XP_009774377.1	1.E-153	95%			
	**XP_009608606.1**	XP_004232135.1	1.E-157	89%	XP_009608606.1	2.E-177	100%	NP_198706.1	4.E-104	63%
					XP_009608607.1	8.E-162	100%			
					XP_009774377.1	1.E-153	95%			
Arabidopsis thaliana	**NP_198706.1**	XP_004232135.1	8.E-105	63%	XP_009608606.1	6.E-104	63%	NP_198706.1	2.E-161	100%
	(AT5G38900.1)				XP_009608607.1	9.E-104	65%			
					XP_009774377.1	1.E-102	65%			
